# Tree growth traits and social status affect the wood density of pioneer species in secondary subtropical forest

**DOI:** 10.1002/ece3.3110

**Published:** 2017-06-14

**Authors:** Lingxiu Chen, Wenhua Xiang, Huili Wu, Pifeng Lei, Shengli Zhang, Shuai Ouyang, Xiangwen Deng, Xi Fang

**Affiliations:** ^1^ Faculty of Life Science and Technology Central South University of Forestry and Technology Changsha Hunan China; ^2^ Huitong National Station for Scientific Observation and Research of Chinese Fir Plantation Ecosystems in Hunan Province Huitong Hunan China

**Keywords:** competitive ability, functional traits, relative growth rate, shade tolerance, tree social status

## Abstract

Wood density (WD) is not only an important parameter to estimate aboveground biomass but also an indicator of timber quality and plant adaptation strategies to stressful conditions (i.e., windthrow, pests, and pathogens). This study had three objectives: (1) to compare WD among seven subtropical tree species; (2) to determine how tree growth traits may influence possible differences in WD between the pioneer and shade‐tolerant species; and (3) to examine whether or not WD differs by tree social status (dominant vs. suppressed trees) within species. To do this, 70 trees were destructively harvested. From each tree, disks at different stem heights were obtained and subjected to a method of stem analysis to measure whole tree level WD. The results showed that WD differed significantly among the seven species (*p *< .001). Their average WD was 0.537 g/cm^3^, ranging from 0.409 g/cm^3^ for *Choerospondias axillaris* to 0.691 g/cm^3^ for *Cyclobalanopsis glauca*. The average WD of the four pioneer species (0.497 ± 0.13 g/cm^3^) was significantly lower (*p *< .01) than that of the three shade‐tolerant species (0.589 ± 0.12 g/cm^3^). The WD of the pioneers had a significant positive correlation with their stem diameter at breast height (DBH), tree height (*H*), and tree age, but WD had a significant negative correlation with relative growth rate (RGR). In contrast, the WD of the shade‐tolerant tree species had no significant relationships with DBH,* H*, tree age, or RGR. The dominant trees of the pioneer species had a higher WD than the suppressed trees, whereas the shade‐tolerant species had a lower WD for dominant trees than the suppressed trees. However, the differences in WD between dominant and suppressed trees were not significant. Taken together, the results suggest that classifying species into pioneer and shade‐tolerant groups to examine the effects of tree growth traits and social status could improve our understanding of intra‐ and interspecific variation in WD among subtropical tree species.

## 1 INTRODUCTION

Woody stem is the most important component of stand biomass (Xiang et al., 2016), carbon (C) stocks (Gao, Taylor, Chen, & Wang, 2016), and net primary production in forest ecosystems (Gower et al., 2001). Woody stem provides several essential functions in trees, namely the mechanical support of aboveground tissues, the storage of water and other resources, and the transport of sap (Chave et al., 2009). Wood density (WD) is a vital woody stem functional trait that is closely related to the quantity and quality of woody products (Acuna & Murphy, 2007; Skovsgaard & Nord‐Larsen, 2011), stand biomass, and C stock estimation (Chave et al., 2009; Plourde, Boukili, & Chazdon, 2015; Wiemann & Williamson, 2002; Zhang et al., 2012) in addition to paleoclimate reconstruction (O'Donnell et al., 2016)—WD is also closely related to the survival and growth rates of species under strong selective pressure in their environment (Falster, 2006; King, Davies, Tan, & Nsm, 2006; Preston, Cornwell, & DeNoyer, 2006). Many studies have reported that WD varies among tree species. Chave et al. (2006) investigated the variation in WD among 2,456 neotropical tree species; they had a mean value of 0.645 g/cm^3^ but had a near 14‐fold range, from 0.11 g/cm^3^ for *Erythrina ulei* Harms. up to 1.39 g/cm^3^ for *Caesalpinia sclerocarpa* Standl. Recently, Yeboah, Burton, Storer, and Opuni‐Frimpong (2014) reported that WD ranged almost threefold, from 0.27 to 0.76 g/cm^3^, for 19 tree species growing in tropical plantations. There are much less data available, however, on WD for subtropical tree species, and where it is found most studies only report the WD of a single species, for example, *Pinus massoniana* (Deng, Zhang, Lei, Xiang, & Yan, 2014; Zhang et al., 2012).

In addition to species differences in WD, several tree growth traits may influence it, namely the diameter at breast height (DBH), tree height (*H*), tree age, and relative growth rate (RGR) (Chave et al., 2006; Kunstler et al., 2016; Raymond & Muneri, 2001; Savva, Koubaa, Tremblay, & Bergeron, 2010; Zhang et al., 2012). Nevertheless, how WD is related to tree growth traits remains controversial. For example, the relationship between WD and DBH was positive for *P. massoniana* (Deng et al., 2014; Zhang et al., 2012), but negative for 27 tree species in a tropical rainforest on Borneo Island (Osunkoya, Sheng, Noraziah, & Norhazlyna, 2007), yet apparently absent for 24‐year‐old Sitka spruce (Livingston, Cameron, Petty, & Lee, 2004). Trees tend to increase their WD to reduce the risk of buckling as they grow taller (O'Brien, Hubbell, Spiro, Condit, & Foster, 1995), but some studies have since shown that trees may increase stem hydraulic conductance for fluid transport to service increasing living biomass when they get taller (Phillips et al., 2003) such that WD is negative correlated with maximum tree height (Thomas, 1996). The relationship between WD and tree age was negative for *Quercus faginea* mature trees (Sousa, Louzada, & Pereira, 2016) and yet positive for European beech (Diaconu, Wassenberg, & Spiecker, 2016). Generally, a negative relationship between WD and RGR has been reported from several tropical forests (Burslem & Whitmore, 2003; Enquist, West, Charnov, & Brown, 1999). In contrast, no relationship was detected between WD and RGR in woody species in New Zealand (Sabrinae et al., 2010) and in a tropical forest in southwest China (Fan, Zhang, Hao, Slik, & Cao, 2012). Unfortunately, similar studies relating WD to tree growth traits are still lacking for subtropical forests.

Light demand is the fundamental characteristic underpinning the classification of “pioneer” (or so‐called shade‐intolerant) versus “shade‐tolerant” tree species. Pioneer tree species tend to grow very well in temporarily lighted conditions, wherein they produce low‐density wood to maximize height growth and stem diameter in early successional habitats (Woodcock & Shier, 2002). These lower WD trees have a higher mass growth rate than their neighbors, often at the expense of greater longevity (Chave et al., 2009; Enquist et al., 1999; King et al., 2006; Plourde et al., 2015). Conversely, shade‐tolerant trees almost always produce higher‐density wood to withstand injury from branch falls from above, as well as potential damage from insect pests and pathogens in the humid understory. As such, these higher WD trees have higher survival rates because they are able to resist damage, disease, and cavitation in the shaded habitats of closed forests (Anten & Schieving, 2010; Putz, Coley, Lu, Montalvo, & Aiello, 1983). Generally, shade‐tolerant tree species usually do have a higher WD than light‐demanding pioneer species (Anten & Schieving, 2010; King et al., 2006; Nock et al., 2009). But upon reaching the canopy, a pioneer tree might then invest more resources into a higher WD rather than maintain rapid growth because its exposure to wind stress is now greater, while the shade‐tolerant tree might continue to produce lower WD wood to increase its trunk area and thus its resistance to bending stresses (Woodcock & Shier, 2002). In subtropical areas, trees species are typically grouped into evergreen conifer, deciduous broadleaved, and evergreen broadleaved species according to their leaf morphological and phenological traits (Iio, Hikosaka, Anten, Nakagawa, & Ito, 2014). The conifer and deciduous trees are pioneer and light‐demanding species that usually occur in the early successional stage, eventually replaced by evergreen broadleaved trees of shade‐tolerant species in the late successional stage (Xiang et al., 2013). Therefore, it is necessary to examine whether the relationship between WD and growth traits may differ between pioneer and shade‐tolerant species.

In forests, where they face strong competition for light, trees become vertically differentiated by their crown or social status, thus leading to both dominant and suppressed trees within species populations. For example, the suppressed trees had a higher WD than did the dominant trees in populations of *P. massoniana* (individuals aged 11–29 years; Deng et al., 2014) and *Picea abies* (Johansson, 1993). Tsoumis and Panagiotidis (1980) reported that for Black pine trees older than 55 years, WD was higher in the suppressed trees than dominant trees in a northern area but this pattern was reversed when in a southern area. Recently, Fajardo (2016) concluded that WD was similar between dominant and suppressed individuals for two temperate species, *Nothofagus betuloides* and *Nothofagus pumilio*. This last result suggests that WD may not be a robust predictor of competitive ability among individuals within species. However, whether this species‐specific pattern extends to other tree species, or varies between different functional types (e.g., pioneer and shade‐tolerant tree species), remains understudied in subtropical forests.

In sum, despite much recent research on WD, how it may vary among tree species in subtropical regions and how it is jointly influenced by light, tree size traits (DBH and *H*), and tree age has been little explored. Here, we investigated inter‐ and intraspecific variation in WD among seven tree species in the subtropical region of Jingzhou County, Hunan Province, southern China. Specifically, this study had three aims: (1) to compare WD among the subtropical tree species; (2) to determine how tree growth traits may influence possible differences in WD detected between pioneer and shade‐tolerant species; and (3) to examine whether WD differs by tree social status for a given species, and whether these differences, if any, may be contrasted for the pioneer and shade‐tolerant species.

## 2 MATERIALS AND METHODS

### 2.1 Site description

This study was carried out in the Paiyashan Forest State Farm (26°10′N; 109°35′E) in Jingzhou County, Hunan Province, China. The farm lies in a subtropical monsoon zone that has a mean annual temperature of 16.7°C and an annual active accumulated temperature of 6,165.8°C. This region receives 1,250 mm of precipitation per year, on average; the mean precipitation is 467.9 mm during the summer (June–August) and 143.8 mm during the winter (December–February). Average daylight totals 1,336.9 hours, with a frost‐free period of 290 days. Annual water surface evaporation is 967.7 mm, on average, while the land evaporation capacity is 603.4 mm (Gou et al., 2017). Elevation ranges between 330 m to 1,075 m. Soils correspond to Alliti‐Udic Ferrosols in the Chinese Soil Taxonomy, or Acrisols in the World Reference Base for Soil Resources (IUSS Working Group WRB 2006). Currently, the main vegetation types in the study region are *Cunninghamia lanceolata* plantations and secondary forest containing many native tree species (Xiang et al., 2016).

### 2.2 Tree sample selection

Seven common tree species were selected for study (Table 1). These included a coniferous tree species (*P. massoniana*; pioneer), three deciduous broadleaved tree species (*Alniphyllum fortunei*,* Choerospondias axillaris*, and *Liquidambar formosana*; pioneer), and three evergreen broadleaved tree species (*Cyclobalanopsis glauca*,* Litsea rotundifolia*, and *Schima superba*; shade‐tolerant). Sampling was carried out in October 2014 before any tree leaves had fallen. After completing the field survey, we selected seven secondary forest stands; in each, we established a 30 × 30 m sampling plot and therein recorded the species names and measured tree diameter at breast height (DBH) and height (*H*) for all stems >1 cm DBH. Based on our plot investigations, 10 trees per species were selected to span the min./max. range of the DBH values (see Xiang et al., 2016). A total of 70 trees were harvested: the DBH of this sample ranged from 2.6 to 52 cm while *H* ranged from 3.5 to 30.2 m.

**Table 1:** Species and family, number of individuals (*N*), and mean values (±*SD*) of diameter at breast height (DBH), tree height (*H*), tree age, relative growth rate (RGR), and wood density (WD) of 70 trees sampled for their wood density determination in Hunan Province, China

Species	Family	*N*	DBH (cm)	*H* (m)	Age (year)	RGR (kg/year)	WD (g/cm^3^_)_
Pioneer species							
* Pinus massoniana*	*Pinaceae*	10	28.4 ± 14.7	16.8 ± 3.6	45.0 ± 15.8	0.13 ± 0.03	0.498 ± 0.09
* Alniphyllum fortunei*	*Styracaceae*	10	21.8 ± 12.4	15.1 ± 5.7	22.2 ± 9.0	0.24 ± 0.05	0.460 ± 0.07
* Choerospondias axillaries*	*Anacardiaceae*	10	13.0 ± 7.0	12.1 ± 2.7	10.7 ± 2.3	0.41 ± 0.10	0.409 ± 0.13
* Liquidambar formosana*	*Hamamelidaceae*	10	27.6 ± 13.9	20.5 ± 7.1	42.0 ± 11.2	0.15 ± 0.02	0.623 ± 0.10
* *Average			22.6 ± 13.5	16.6 ± 5.9	29.7 ± 17.7^a^	0.24 ± 0.13^a^	0.497 ± 0.13^a^
Shade‐tolerant species							
* Cyclobalanopsis glauca*	*Fagaceae*	10	27.8 ± 14.7	17.5 ± 4.3	50.4 ± 14.0	0.12 ± 0.03	0.691 ± 0.12
* Schima superba*	*Theaceae*	10	15.7 ± 8.4	12.9 ± 3.9	42.8 ± 15.6	0.13 ± 0.03	0.592 ± 0.11
* Litsea rotundifolia*	*Lauraceae*	10	22.9 ± 14.7	13.2 ± 5.4	47.9 ± 18.4	0.13 ± 0.04	0.485 ± 0.04
* *Average			22.7 ± 13.5	14.9 ± 5.1	47.9 ± 18.4^b^	0.13 ± 0.04^b^	0.589 ± 0.12^b^
All species			22.7 ± 13.4	15.9 ± 5.64	37.0 ± 19.9	0.19 ± 0.11	0.537 ± 0.13

Letters (a and b) indicate a significant difference at *p *<* *.01 level between the pioneer and shade‐tolerant species.

### 2.3 Woody density measurement

To determine its WD, a stem was first sectioned at 1.3‐m and then at 1‐m or 2‐m intervals for those trees with *H* < 10 m or *H* > 10 m, respectively. The fresh weight and diameter of each stem section were measured after the bark had been carefully removed. A 3–5‐cm‐thick disk was cut from the bottom of each section and its weight immediately measured. The stem disks at the ground surface were cut to produce a smooth surface clearly showing the tree ring boundaries. After weighing in situ, each disk was put into a plastic bag to prevent water losses. These bagged disks were transported to the laboratory to measure tree age—by counting the ring numbers—and to determine their disk volume by the water displacement method. We calculated a relative growth rate (RGR) based upon the whole tree biomass and tree age as determined by the rings of the stem disk at the ground surface. The following formula was used for RGR:

RGR=(lnM)/dt

where *M* is the biomass of a harvested tree and *dt* is the tree's age.

Finally, all disks were oven‐dried at 105°C to a constant weight to determine each disk's dry weight. The WD for each disk was calculated by dividing its dry mass by its fresh volume. For a given tree, WD was a weighted average calculated from each disk's WD (using the dry mass of each section as the weighting factor to arrive at a value of WD for the whole tree).

### 2.4 Data analysis

One‐way analysis of variance (ANOVA) and the Tukey honest significant difference (HSD) test were used to detect significant differences in WD among the seven species. ANOVAs were likewise carried out on DBH, *H*, tree age, RGR, and WD to detect differences among the species of pioneer and shade‐tolerant trees. The relationships of WD against DBH, *H*, and tree age were examined by using linear regressions separately performed for the pioneer and shade‐tolerant tree species. Linear regression was also used to evaluate the relationships of RGR against WD and tree size (DBH and *H*) for the pioneer species and shade‐tolerant species. Finally, to explore the influence of tree social status of each species on WD, the dominant trees were determined by the height at which they reached the forest canopy; the suppressed trees were those of similar age but having a height 20% lower than that of the dominant individuals (Table 2). For each species, a *t*‐test examined the difference in WD between the dominant and suppressed individuals. Statistical significance was set at a level of α = 0.05. All analyses were carried out in the statistical software platform, R, version 3.2.0 (R Development Core Team 2015).

**Table 2:** Number (*N*) and mean values (±*SE*) of tree age, diameter at breast height (DBH), tree height (*H*), and wood density (WD) for dominant and suppressed individuals of the pioneer and shade‐tolerant species

	Pioneer		Shade‐tolerant	
	Dominant	Suppressed	Dominant	Suppressed
*N*	11	11	9	9
Age (year)	28.0 ± 16.2	27.3 ± 15.7	56.8 ± 11.6	55.3 ± 12.1
DBH (cm)	26.4 ± 10.7	19.3 ± 13.6	32.4 ± 11.4	20.7 ± 8.7
*H* (m)	19.2 ± 6.4	15.6 ± 5.7	17.9 ± 4.0	14.8 ± 3.9
WD (g/cm^3^_)_	0.52 ± 0.12	0.43 ± 0.12	0.53 ± 0.11	0.62 ± 0.14

## 3 RESULTS

### 3.1 Variation in wood density among the tree species

**Figure 1:**
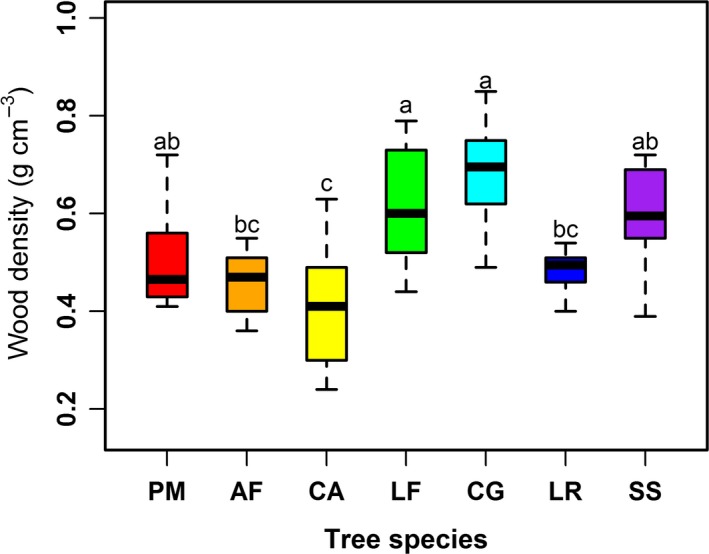
Species comparisons of stem wood density (WD) for *Pinus massoniana* (PM), *Alniphyllum fortunei* (AF), *Choerospondias axillaris* (CA), *Liquidambar formosana* (LF), *Cyclobalanopsis glauca* (CG), *Litsea rotundifolia* (LR), and *Schima superba* (SS). Box plots show the range, median, and 25% and 75% quartiles of species‐specific average wood density in Hunan Province, China. Different letters indicate a significant difference in wood density among the species at *p *<* *.01

Wood density (WD) had a mean value of 0.537 g/cm^3^ for all trees of the seven species. However, WD differed significantly among the seven species (*p *< .001, *F*_6, 63_ = 10.45) (Figure 1). Average WD was highest in *C. glauca* (0.691 g/cm^3^), closely followed by *L. formosana* and *S. superba*, and then by *P. massoniana*,* L. rotundifolia*, and *A. fortune*; it was lowest in *C. axillaris* (0.409 g/cm^3^) (Table 1). In general, the average WD for the four pioneer tree species (0.497 ± 0.13 g/cm^3^) was significantly lower (*p* < .01) than that of the three shade‐tolerant tree species (0.589 ± 0.12 g/cm^3^) (Table. 1).

### 3.2 Relationship between wood density and the growth traits

**Figure 2:**
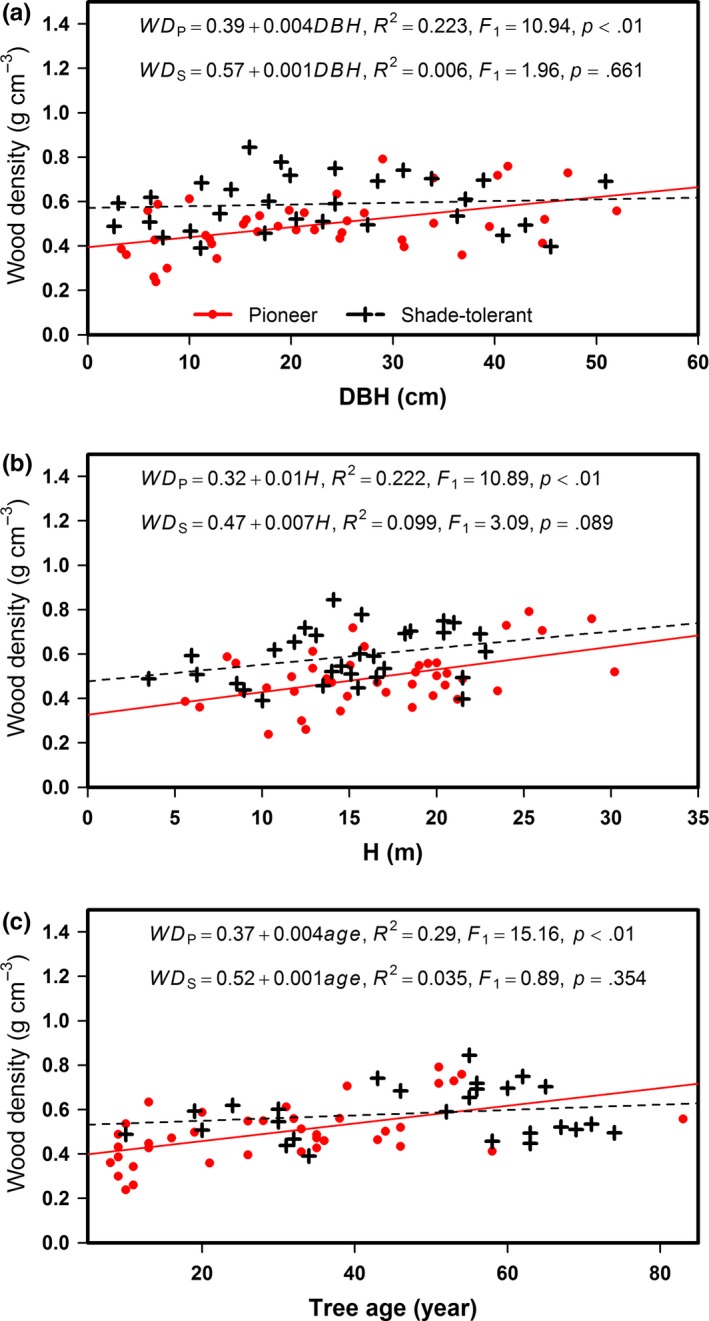
Relationships between stem wood density (WD) and (a) stem diameter at breast height (DBH), (b) individual tree height (*H*), and (c) tree age. Red solid line, red full dots, and the equation WD_p_ represent the pioneer tree species. Black dashed line, black plus signs, and the equation WD_s_ represent the shade‐tolerant tree species

**Figure 3:**
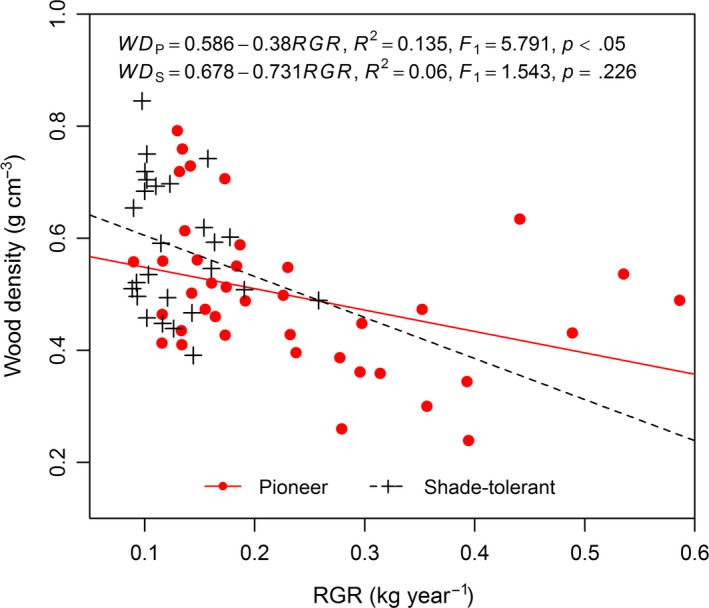
Relationships between stem wood density (WD) and relative growth rate (RGR). Red solid line, red full dots, and the equation WD_p_ represent the pioneer tree species. Black dashed line, black plus signs, and the equation WD_s_ represent the shade‐tolerant tree species

**Figure 4:**
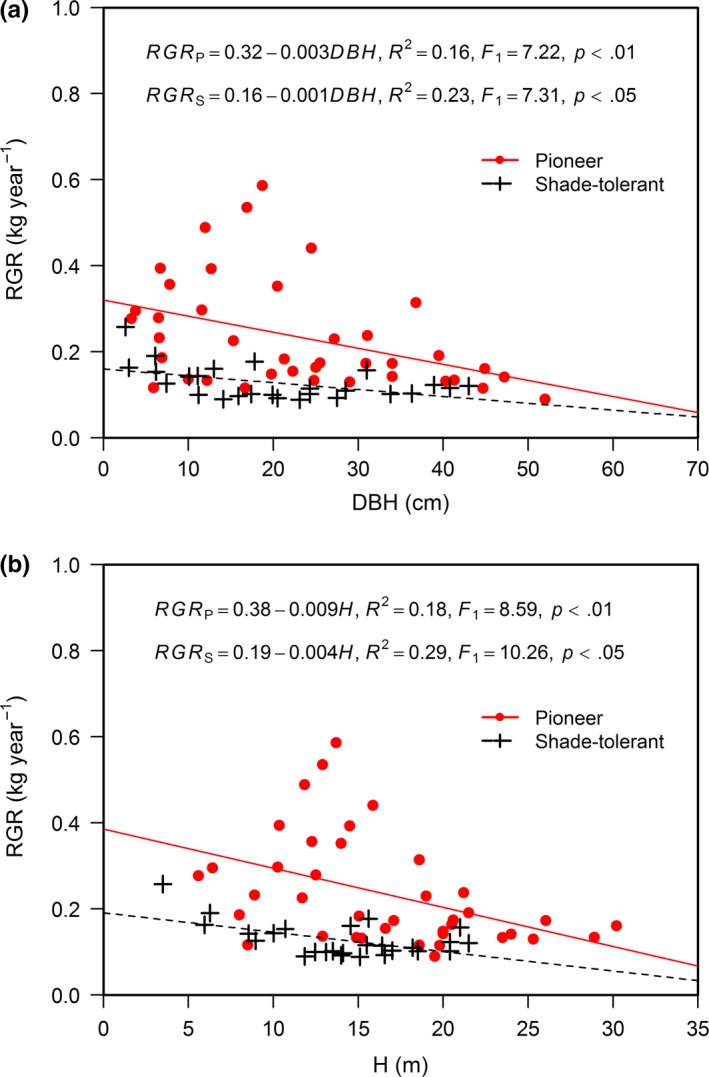
Relationships between relative growth rate (RGR) and tree size (DBH and *H*, respectively, for stem diameter and height). Red solid line, red full dots, and the equation WD_p_ represent the pioneer tree species. Black dashed line, black plus signs, and the equation WD_s_ represent the shade‐tolerant tree species

**Figure A1:**
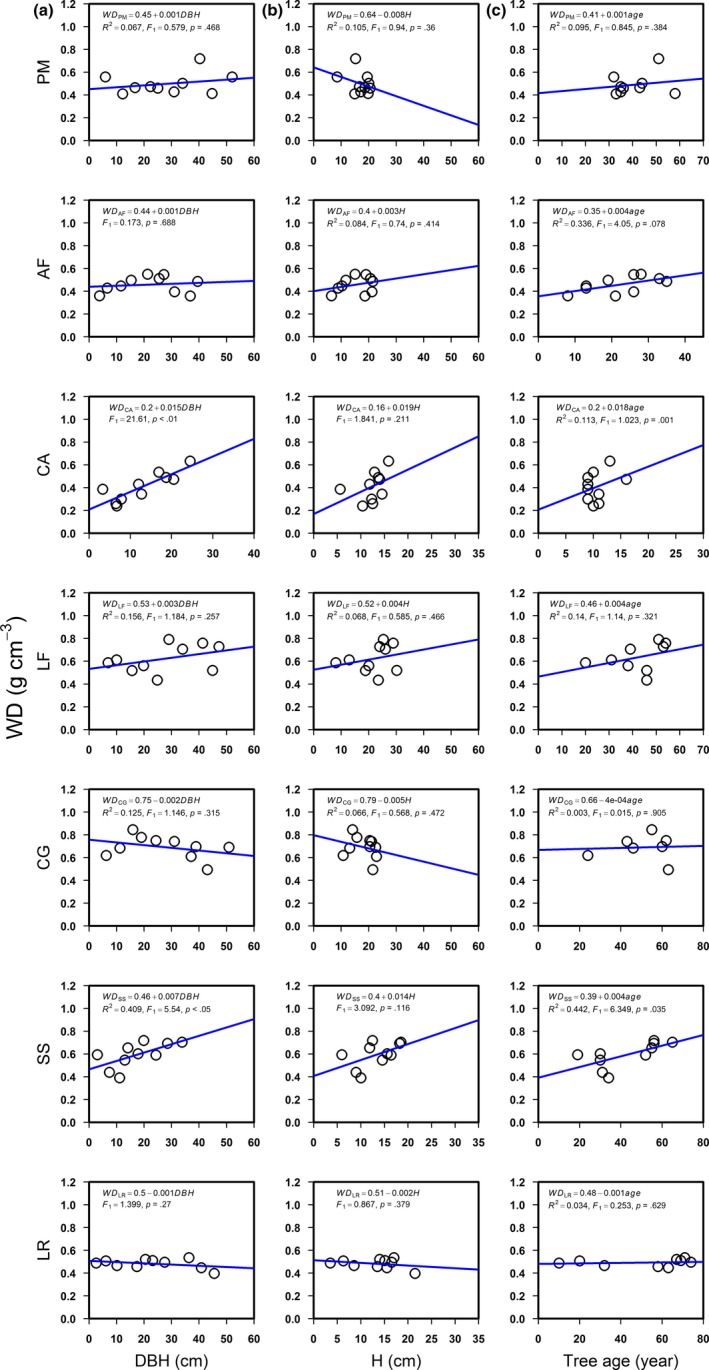
Linear regression relationships between wood density (WD) and diameter at breast height (DBH) (panel column a), tree height (*H*) (panel column b), and tree age (panel column c) of each species. Species abbreviations: PM (*Pinus massoniana*), AF (*Alniphyllum fortunei*), CA (*Choerospondias axillaris*), LF (*Liquidambar formosana*), CG (*Cyclobalanopsis glauca*), LR (*Litsea rotundifolia*), and SS (*Schima superba*)

**Figure A2:**
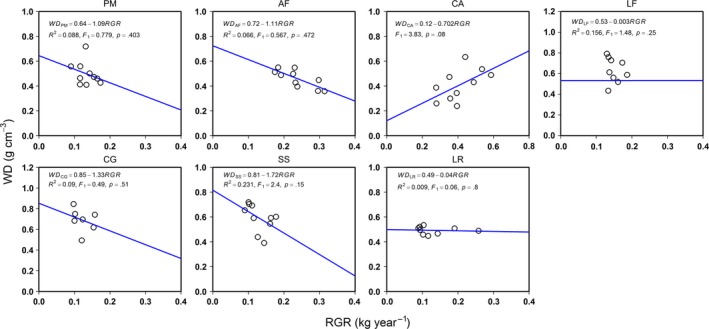
Linear regression relationships between wood density (WD) and relative growth rate (RGR) of each species. Species abbreviations: PM (*Pinus massoniana*), AF (*Alniphyllum fortunei*), CA (*Choerospondias axillaris*), LF (*Liquidambar formosana*), CG (*Cyclobalanopsis glauca*), LR (*Litsea rotundifolia*), and SS (*Schima superba*)

**Figure A3:**
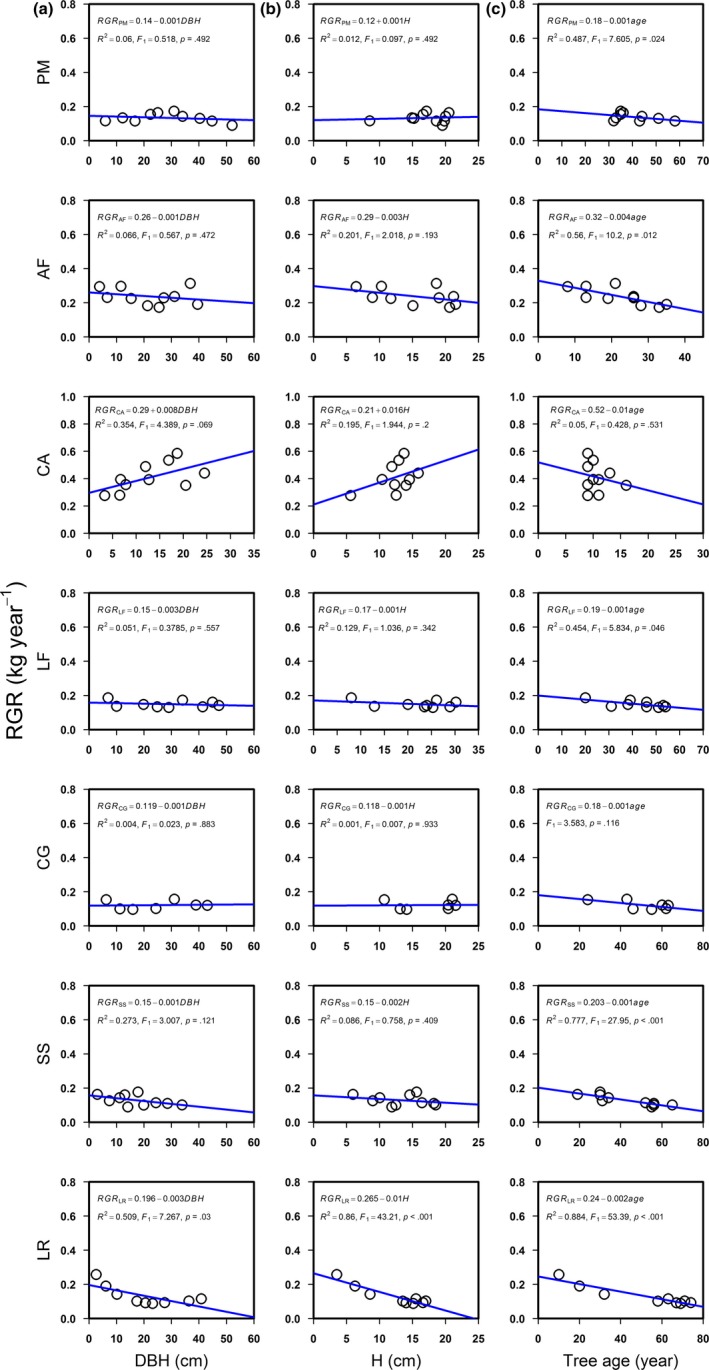
Linear regression relationships between relative growth rate (RGR) and diameter at breast height (DBH) (panel column a), tree height (*H*) (panel column b), and tree age (panel column c) of each species. Species abbreviations: PM (*Pinus massoniana*), AF (*Alniphyllum fortunei*), CA (*Choerospondias axillaris*), LF (*Liquidambar formosana*), CG (*Cyclobalanopsis glauca*), LR (*Litsea rotundifolia*), and SS (*Schima superba*)

Average tree age and relative growth rate (RGR) differed significantly between the pioneer and shade‐tolerant species (*p *< .01), whereas no significant differences in average DBH and *H* were found between the pioneer and shade‐tolerant species (Table 1). Negative relationships of WD against tree size (DBH and *H*) and tree age were significant for the pioneer tree species (*p *<* *.01), although not significant for the shade‐tolerant species (Figure 2, Figure A1). As RGR increased the WD of the pioneer species significantly decreased (*p *<* *.05), but this linear trend was not significant for the shade‐tolerant species (Figure 3, Figure A2). RGR was significantly and negatively correlated with DBH and *H* (*p *< .05) (Figure 4, Figure A3), so the effects of RGR on WD are likely, in part, attributed to tree size.

### 3.3 Differences in wood density between tree social status

**Figure 5:**
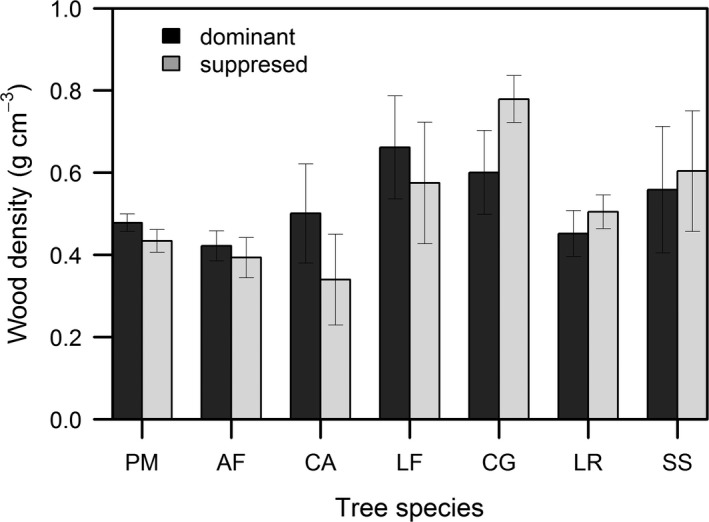
Comparison of mean (±*SE*) stem wood density between dominant and suppressed individuals within the seven tree species. Species abbreviations for each life‐history group: pioneers: PM (*Pinus massoniana*), AF (*Alniphyllum fortunei*), CA (*Choerospondias axillaris*), and LF (*Liquidambar formosana*); shade tolerants: CG (*Cyclobalanopsis glauca*), LR (*Litsea rotundifolia*), and SS (*Schima superba*)

Pioneer species had a higher WD in their dominant than suppressed trees while the shade‐tolerant species exhibited the opposite pattern. But the differences in WD between the dominant and suppressed trees were not significant for any tree species (Figure 5).

## 4 DISCUSSION

### 4.1 Variation in wood density among the tree species

In this study, WD significantly differed among the seven species. This is an expected result consistent with studies carried out elsewhere. For instance, WD differed across 335 tree species in a Panamanian moist forest (Hietz, Valencia, & Wright, 2013) and globally among tropical tree species (Lewis et al., 2009). Importantly, the WD values between 0.409 and 0.691 g/cm^3^ from this present study are within the range (0.11 to 1.39 g/cm^3^) reported for 2,456 tropical forest tree species (Chave et al., 2006). However, the average WD (0.537 g/cm^3^) in our study was lower than that (0.570 g/cm^3^) found for trees from two neotropical rain forests (Hietz et al., 2013). At the species level, the WD for *P. massoniana* (0.498 g/cm^3^) in this study was higher than that found by Zhang et al. (2012) (0.484 g/cm^3^) and Deng et al. (2014) (0.477 g/cm^3^). The differences in WD between our study and other studies may be due to aspects of the growing environment (phenotypic factors) such as light, soil fertility, and precipitation (Baker et al., 2004; Kunstler et al., 2016; Maharjan et al., 2011; Muller‐Landau, 2012; Raymond & Muneri, 2001), genus identity (Chave et al., 2006), as well as properties of the fiber lumen and wall (Berkooz, Komargodski, & Reichmann, 2015; Ziemińska, Butler, Gleason, Wright, & Westoby, 2013).

As a group, the shade‐tolerant tree species had a significantly higher WD than did the pioneer species (Table 1). Previous studies also reported that shade‐tolerant species tend to have a much higher WD than do pioneer species (King et al., 2006; Ramananantoandro, Ramanakoto, Rajoelison, Randriamboavonjy, & Rafidimanantsoa, 2016). One explanation is that pioneer species produce a low WD to grow taller than their neighbors, thereby acquiring more resources (i.e., light) quickly (Woodcock & Shier, 2002), thus enabling them to grow faster but at the cost of a high WD (Thomas & Malczewski, 2007). Conversely, shade‐tolerant tree species occur in late successional stages where the forest canopy is closed, growing mostly under shady conditions (Anten & Schieving, 2010). Shade‐tolerant species will acquire less light, leading to a slower growth rate, thus enabling them to produce a higher WD to better prevent disease and damage than can the pioneer tree species (Putz et al., 1983). To date, most studies of the variation in WD were conducted in tropical forests, but few studies have been conducted in subtropical forests. Therefore, this study will help fill the gap in knowledge of the variation in WD between pioneer and shade‐tolerant species.

### 4.2 Effects of tree growth traits on the variation in wood density

In this study, the positive relationships of WD against key growth traits (DBH and *H*) and tree age were significant for the pioneer trees, but they were not significant for shade‐tolerant trees. These positive relationships are consistent with other field reports (Henry et al., 2010; Lida & Kohyama, 2012; Zhang et al., 2012). For example, the WD of Malaysian rainforest trees showed a positive relationship with *H* at a standardized stem diameter (Lida & Kohyama, 2012). This could arise if denser wood is stronger and stiffer, and allows trees to produce more slender stems (Poorter, Lianes, las Heras, & Zavala, 2012). DBH, *H*, and tree age are generally considered important tree growth traits, especially if those individuals with a larger DBH and *H* are also older. According to our results their relationship to WD was significant only for the pioneer species group. A plausible explanation is as follows: young trees that can grow vigorously in canopy gap areas, that is, under high light conditions, “face a high selective pressure to quickly grow in height or else they would be out‐competed by faster growers” (Woodcock & Shier, 2002), which comes at a structural cost in the form of low‐density wood due to their initial fast growth; however, as juveniles grow taller, mature, and age, producing high‐density wood would confer unto them greater structural support and mechanical stability to enhance their lifetime reproductive output (Fajardo, 2016; Henry et al., 2010; Rueda, 1993).

As expected, the result that pioneer tree species had a significantly higher RGR than shade‐tolerant species agrees with many field studies (e.g., Ruizrobleto & Villar, 2005; Souza & Válio, 2003). In our study, the WD of the pioneer species was significantly and negatively correlated with their RGR, but not so for the shade‐tolerant species. The relationships between WD and RGR reported by some studies are quite different, however. Although WD and RGR were negatively correlated in many tropical woody species (Burslem & Whitmore, 2003; Enquist et al., 1999; Hoeber, Leuschner, Köhler, Arias‐Aguilar, & Schuldt, 2014; Muller‐Landau, 2012), there was no relationship detected between WD and RGR in temperate tree species (Debell, Singleton, Gartner, & Marshall, 2004; Sabrinae et al., 2010) and some Asian tropical tree species (Fan et al., 2012). Comparatively, there has been disproportionately little research conducted into differing relationships between WD and RGR variables between pioneer and shade‐tolerant species.

The apparent divergence in patterns between the pioneer and shade‐tolerant species may relate to the minimum support required by a tree subject to no stresses other than its own weight (King et al., 2006). Previous studies have suggested that both WD and support costs are correlated with the degree of tree species shade tolerance (Givnish, 1988; King, 1994; King et al., 2006). Photosynthetic adaptations for high productivity under lit conditions appear to be associated with low support costs (Coley, 1983), and low support costs are now recognized as part of a larger suite of adaptations for rapid growth that tend to reduce WD (Kitajima, 1994). Thus, all else being equal, for pioneer species their WD decreases as RGR increases because lower support costs are invested toward the stem and more to the crown to enhance light interception and growth rate (King et al., 2006). Nonetheless, another possibility exists, by which a lower WD may lead to an increase in the relative proportion of stem conduit to meet water and nutrients required by the processes of photosynthesis, transpiration, and growth (Chave et al., 2009; Missio et al., 2016). The pioneer species—due to their initial fast growth—inherently have greater potential for variation in WD. Hence, the value of RGR could significantly influence the WD of pioneer species. Saner et al. (2016) found that with increasing light pioneer species had increased growth and decreased WD, whereas the shade‐tolerant species were not as sensitive in their response to light changes. Tree age may be another factor by which RGR can influence WD. In this study, the average tree age was 29.66 years for the pioneer species and 47.88 years for the shade‐tolerant species. When the average tree age of shade‐tolerant species exceeds 30 years, it may lead to no significant relationship between WD and RGR (Debell et al., 2004). In addition, both WD and RGR were significantly associated with tree size in this study; this may explain party why RGR was related to WD for the pioneer species. Put shortly, because the WD of the pioneer and shade‐tolerant species showed different relationships with the growth traits (DBH, *H*, tree age, and RGR), it suggests a new research direction toward resolving the current inconsistencies concerning these relationships in forest trees.

### 4.3 Interspecific variation in wood density between tree social status

In this study, the WD of the four pioneer tree species (*P. massoniana*,* A. fortunei*,* C. axillaris*, and *L. formosana*) were higher in the dominant than in the suppressed individuals. This result is perhaps best explained by the fact that, at least early in their lifetime, pioneers must invest in tree height (*H*) growth to continually acquire more light resources, thereby sacrificing gains in mechanical strength and thus producing a lower WD. But upon reaching the canopy, as wind stress likely increases, further gains in height seem no longer important or perhaps even necessary. Instead, the priority for these now‐adult trees is to invest to strengthen their ability to resist windthrow, which could be achieved by producing a higher WD (Hietz et al., 2013). In this manner, also argued for by Fajardo (2016), pioneer trees should have a lower WD early in ontogeny as seedlings, saplings, and poles, while in the later life stages they increase in WD to build and ensure greater mechanical stability in the forest environment.

Counter to this pattern, the WD of the shade‐tolerant tree species (*C. glauca*,* L. rotundifolia*, and *S. superba*) was higher in the suppressed than dominant individuals. The simplest explanation for this result is that juvenile trees of shade‐tolerant species have biophysical adaptions to persist under the closed canopy, where they can survive decades under low light conditions by producing higher WD to withstand branch falls from above and to better resist pests favoured by a humid environment. Upon reaching the canopy, the previously suppressed shade‐tolerant trees might then increase in trunk girth to strengthen their resistance to bending and thus avoid mechanical failure, but this would entail a cost in protective chemical strength and would unavoidably result in a lower WD during the adult stage (Fajardo, 2016; Woodcock & Shier, 2002). That the difference in WDs between dominant and suppressed trees was not significant in our study is a result similar to that reported by Fajardo (2016). This could be attributed to the low competition intensity for light between dominant and suppressed trees of a given species.

## 5 CONCLUSIONS

Subtropical tree species in southern China showed significant variation in their wood density. There was a significant and positive relationship of WD as a function of increasing DBH, *H*, and tree age for the group of pioneer tree species, but such relationships were not found for the shade‐tolerant group. RGR was significantly and negatively correlated with WD for the pioneer species but not for the shade‐tolerant species. In the four pioneers (i.e., *P. massoniana*,* A. fortunei*,* C. axillaris*, and *L. formosana*), the WD of the dominant individuals was higher than that of the suppressed ones, while this pattern was reversed in the shade‐tolerant species. However, the differences in WD between the dominant and suppressed trees were not significant for any species. These results imply that the influence of tree growth traits on WD is more pronounced for pioneer than shade‐tolerant tree species, at least in this part of subtropical China.

## AUTHOR CONTRIBUTIONS

WX and LP conceived the idea and took part in study design; LC, SZ, SO, XD, and XF with support from WX performed data collection and analysis; LC, WX, and HW wrote the manuscript.

## CONFLICT OF INTEREST

None declared.
